# Exploring the epigenetic regulation of telomerase reverse transcriptase (TERT) in human cancer cell lines

**DOI:** 10.1002/1878-0261.12798

**Published:** 2020-09-19

**Authors:** Brittany A. McKelvey, Martha A. Zeiger, Christopher B. Umbricht

**Affiliations:** ^1^ Department of Surgery Johns Hopkins University Baltimore MD USA; ^2^ Department of Molecular Biology and Genetics Johns Hopkins University Baltimore MD USA; ^3^ Surgical Oncology Program National Cancer Institute National Institutes of Health Bethesda MD USA; ^4^ Department of Oncology Johns Hopkins University Baltimore MD USA; ^5^ Department of Pathology Johns Hopkins University Baltimore MD USA

**Keywords:** epigenetic regulation, monoallelic gene expression, monoallelic methylation, telomerase reverse transcriptase, *TERT* promoter methylation, *TERT* promoter mutation

## Abstract

Telomerase regulation, including *TERT* promoter methylation, has been of long‐standing interest to cancer biologists. Rowland *et al*. have now vastly expanded their ongoing characterization of *TERT* promoter methylation in cancer cells, analyzing the methylation patterns of 833 cell lines from 23 human cancers. They document a highly conserved pattern of hypomethylation around the proximal promoter, as well as a more heterogeneous region of hypermethylation further upstream, both associated with active *TERT* expression in cancer cells. They further describe the interplay between activating *TERT* promoter mutations and allelic methylation and transcription patterns. This valuable dataset represents the most extensive characterization of *TERT* promoter methylation in cancer cells to date and will help guide the future study of transcriptional regulation of telomerase.

Comment on: https://doi.org/10.1002/1878‐0261.12786

## Graphical abstract

Allelic *TERT* promoter methylation and transcription scenarios in cancer:

1) biallelic wt; 2) monoallelic mutant; 3) monoallelic wt; 4) amplified mutant.

The transcriptional start site (angled arrow) and proximal (light blue) and distal (dark blue) *TERT* promoter regions with allele‐specific transcription levels are depicted. Unmethylated cytosine‐guanine dinucleotide (CpG) is green; methylated CpG is red.

C or T indicates a C‐>T *TERT* promoter mutation site.

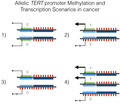

**Abbreviations**
BAE: biallelic expression
bp: base pair
C‐>T: cytosine to thymidine
CpG: cytosine‐guanine dinucleotide
MAE: monoallelic expression
MSP: methylation‐specific PCR
nCATS: nanopore Cas9‐targeted sequencing
PCR: polymerase chain reaction
SNP: single‐nucleotide polymorphism
TERT: telomerase reverse transcriptase
WT: wild‐type

## 1 Introduction

Telomerase maintains the length of chromosomes by adding (TTAGGG)_n_ hexamer repeats in a dynamic equilibrium with chromosomal end loss during DNA replication. The catalytic subunit, telomerase reverse transcriptase (*TERT*), is transcriptionally active in germline, stem, and cancer cells, but silenced in somatic cells [1], making it a gatekeeper of cellular immortalization while maintaining chromosomal integrity.

Telomerase activation is regulated by the interplay of multiple factors, including genetic changes, such as promoter mutations and copy number variation; epigenetic changes, such as promoter methylation and histone marks; transcription factor binding; and alternative splicing of *TERT* [2, 3, 4]. The study of *TERT* promoter methylation has been complicated by reports of *hyper*methylation of the *TERT* promoter in cancer, seemingly contradicting the canonical model of promoter methylation causing transcriptional silencing by inhibiting transcriptional activator binding. This was due in part to the limited number of *TERT* promoter cytosine‐guanine dinucleotide (CpGs) interrogated by methylation‐specific PCR (MSP) and available methylation array platforms.

With the increasing availability of bisulfite sequencing, a more complete picture of *TERT* methylation patterns is emerging, albeit with the limitation that bisulfite treatment, which converts unmethylated cytosine to uracil/thymidine, also obscures the presence of the two most common *TERT* promoter‐activating mutations [both cytosine to thymidine (C‐>T)]. This in turn makes it challenging to directly link allele‐specific methylation patterns to the mutant allele. This issue can sometimes be addressed by finding fortuitously located single‐nucleotide polymorphisms (SNP)s, but can be avoided entirely with novel long‐read sequencing methods such as nanopore Cas9‐targeted sequencing [5], as this technology does not require bisulfite treatment to identify methylcytosines, but it will take time for these data to accrue in public datasets.

Despite these challenges, after analyzing bisulfite sequencing data currently available in the Broad Institute’s Cancer Cell Line Encyclopedia [6], Rowland *et al*. [7] present a comprehensive catalog of *TERT* promoter methylation in 833 human cancer cell lines from 23 different cancer types. This dramatically expands upon the long‐standing work of the Cech laboratory characterizing and understanding epigenetic regulation of the *TERT* promoter, covering a 2788‐bp region from the upstream promoter to the initial *TERT* exons. Methylation was also analyzed in the context of allelic expression, that is, monoallelic (MAE) *TERT* wt, MAE *TERT* mutant, and biallelic (BAE) *TERT* wt, in 107 cell lines previously classified based upon their allelic expression [8].

## 2 Key findings

Telomerase‐expressing cancer cell lines consistently exhibited hypermethylation of the upstream promoter and hypomethylation of the proximal promoter near the transcriptional start site and into exon 1.

Compared to MAE lines, BAE cell lines showed decreased methylation of the proximal promoter. Furthermore, MAE *TERT* wt and mutant cell lines exhibited allele‐specific demethylation associated with active *TERT* transcription. These findings were also confirmed by bisulfite conversion cloning of cell lines representative of the three allelic expression classes, MAE wt, MAE mutant, and BAE wt.

The significance of these methylation patterns was further supported by chromatin immunoprecipitation experiments, which showed the expected association of the demethylated proximal promoter with histone marks of active transcription.

Taking advantage of a SNP located in exon 2, long‐range MSP confirmed the association of a demethylated proximal promoter with the actively transcribed allele.

## 3 Conclusions

This comprehensive analysis makes a strong case for reconciling the previously diverging interpretations of the role of *TERT* promoter methylation patterns in cancer. Active telomerase transcription is associated with both hypermethylation of some upstream regions of the promoter, including the prognostically useful upstream CpG marker at cg11625995, and consistent hypomethylation of the proximal promoter. Both patterns are present in a large number of cancer subtypes. Importantly, the proximal promoter methylation pattern is detectable in the allele‐specific analysis, with consistent histone marks and allele‐specific transcription in the cell lines examined.

## 4 Outlook

Scientific progress raises new questions and reframes old ones. As noted by the authors, it remains unclear which of the above findings are necessary and/or sufficient for transcriptional activation. This is because stem cells are largely unmethylated across the promoter and express telomerase, while differentiated normal tissues are similarly unmethylated, but do not express telomerase. It is also unclear what role the upstream hypermethylation frequently observed in cancer cells plays. It may prevent binding of transcriptional repressors in cancer, but it is clearly not necessary for *TERT* silencing in differentiated normal tissue, which lacks such hypermethylation.

The answer may depend on context, where regulatory programs depend on the physiological state of a cell. Processes of malignant transformation may activate transcriptional repressors not expressed in stem cells, creating the need to block their *TERT* promoter binding sites in transformed cells, but not in untransformed stem cells not expressing them.

Overall, this also illustrates a gap in our understanding of telomerase regulation in physiological states, since almost all work is performed in cancer cells or transformed cell lines. Work in normal cells poses significant technical difficulties, but given the unavoidable involvement of telomerase in the establishment of cell lines, it will remain difficult to extrapolate results to the untransformed state. Hopefully, experiments with stem cells, short‐term cultures, and cell organoids will prove informative.

Another open question concerns the role of *TERT* promoter mutations or *TERT* amplifications, with their associated increase in telomerase expression, often present in aggressive or advanced cancer. These are clearly unnecessary for immortalization or even malignant transformation, since most cancers lack these structural changes. The high telomerase levels found in the original telomerase model organism [9], *Tetrahymena thermophila*, with its physiological chromosomal fragmentation, may point to a role for high levels of telomerase in stabilizing the late‐stage cancer genome.

Finally, this dataset clearly sets the stage for promising future investigations. Inspection of the reported methylation patterns shows several unexplained distinctive foci of relative hypomethylation within the larger upstream hypermethylation, the most prominent occurring between the distal E‐box and the biomarker cg11625005. Investigating a similar focal hypomethylation in thyroid cancer cell lines, we discovered a previously unpublished binding site for the goosecoid homeobox protein (*GSC*), a known oncogene [10]. In summary, identifying additional regulatory elements and examining their role in telomerase gene expression will certainly improve our understanding of this key player in carcinogenesis.

## Conflict of interest

The authors declare no conflict of interest.

## Author contributions

BAM, MAZ, and CBU contributed equally to the manuscript.
